# Edoxaban, a direct oral factor Xa inhibitor, ameliorates coagulation, microvascular thrombus formation, and acute liver injury in a lipopolysaccharide-induced coagulopathy model in rats

**DOI:** 10.1007/s11239-021-02381-y

**Published:** 2021-02-03

**Authors:** Yoshiyuki Morishima, Tomoko Shibutani, Kengo Noguchi, Yusuke Ito, Yuko Honda

**Affiliations:** 1 Medical Science Department, Daiichi Sankyo Co., Ltd., 3-5-1 Nihonbashi Honcho, Chuo-ku, Tokyo, 103-8426 Japan; 2 Translational Research Department, Daiichi Sankyo RD Novare Co., Ltd, Tokyo, 134-8630 Japan; 3 Pharmacovigilance Department, Daiichi Sankyo Co., Ltd., Tokyo, 103-8426 Japan; 4 Specialty Medicine Research Laboratories II, Daiichi Sankyo Co., Ltd., Tokyo, 140-8710 Japan; 5 Biological Research Laboratories, Daiichi Sankyo Co., Ltd., Tokyo, 103-8426 Japan

**Keywords:** Lipopolysaccharide, Coagulation, Microvascular thrombus, Organ damage, Inflammatory cytokine, Edoxaban

## Abstract

Infection increases the risk of thrombosis through the activation of inflammation and coagulation. Edoxaban, a direct oral factor Xa inhibitor, is used for the prevention and treatment of thrombotic diseases. The aim of this study was to determine the effects of edoxaban on microvascular thrombus formation in a rat model of lipopolysaccharide (LPS)-induced coagulopathy. Rats were intravenously injected with 7.5 mg/kg of LPS (*Escherichia coli* 055:B5). Immediately after LPS injection, the rats were treated with subcutaneous injection of edoxaban. At 2 and 6 h after the injection of LPS, biomarkers of coagulation and organ damages and inflammatory cytokines were measured. Microvascular thrombus formation in organs was evaluated using ^125^I-fibrinogen (human) or by the pathological analysis. Mortality was examined 24 h after LPS injection. After the injection of LPS, D-dimer and thrombin-antithrombin complex increased and platelet numbers decreased, indicating the activation of coagulation. Microvascular thrombi were found in the liver. Markers of liver injury (aspartate aminotransferase and alanine aminotransferase) also increased. Treatment with edoxaban attenuated the changes in the coagulation markers and microvascular thrombus formation in the liver. Edoxaban suppressed the increase in the liver injury markers and reduced the mortality. Edoxaban did not affect the levels of inflammatory cytokines. In conclusions, edoxaban significantly inhibited the activation of coagulation, the formation of microvascular thrombus in the liver and the liver damage, and reduced mortality in rats injected with LPS. These results suggest that the FXa inhibition by edoxaban might be a beneficial therapy for the management of infection-associated thrombosis.

## Highlights

- LPS activates coagulation and induces microvascular thrombus formation in the liver.
- LPS promotes increase in liver injury markers and induces death.
- Edoxaban attenuates coagulation activation and microvascular thrombus formation.
- Edoxaban suppresses the increase in the liver injury markers and reduces mortality.

## Introduction

Infection is known as one of the risk factors of thromboembolic diseases such as venous thrombosis (deep vein thrombosis and pulmonary embolism) and arterial thrombosis (myocardial infarction and stroke) [1–3]. Although there are several types of pathogens (bacteria and viruses) and symptoms (pneumonia, urinary tract infection, and systemic infection etc.), all types of infections can elevate the risk of thrombosis. In general, the activation of coagulation is beneficial for infections to limit pathogen dissemination and support pathogen killing. However, over-activation of coagulation can lead to thrombosis and result in poor outcome [4].

To investigate the pathological mechanisms and potential drugs for the prevention and/or treatment of infection-associated thrombosis, animal models have been established. Typical animal models are induced by intravenous or intraperitoneal injection of a single product derived from pathogens. As the single product, lipopolysaccharide (LPS) is most frequently used. LPS is a major component of the outer cell membrane of gram-negative bacteria like *Escherichia coli* and is recognized by host cells [5]. Binding of LPS to host cells such as monocytes/macrophages, platelets and endothelial cells results in a procoagulant state through directly activating pro-inflammatory responses simultaneously with coagulation factors like monocyte-derived tissue factor [6]. Inflammatory cytokines produced in the LPS model upregulate the coagulation factors such as tissue factor [7].

Effects of anticoagulants on infection-associated thrombosis using LPS were determined in several studies, in which low molecular weight heparin (LMWH) [8], tissue factor pathway inhibitor (TFPI) [9], and hirudin [6] significantly inhibit the formation of microvascular thrombus in several organs like the liver and kidneys. Recently, direct oral anticoagulants (DOACs) are widely used for the prevention of stroke and systemic embolism in patients with nonvalvular atrial fibrillation, the prophylaxis of venous thromboembolism (VTE) after orthopedic surgery, and the treatment of VTE and the prevention of recurrent VTE. DOACs directly inhibit a single activated coagulation factor, factor Xa (FXa) or thrombin, and significantly suppress the thrombosis in animal models [10–13]. However, the effects of DOACs on the infection-associated thrombosis is unclear.

In this study, we evaluated the effects of edoxaban, which is one of the DOACs with the mechanism of the direct inhibition of FXa, on blood coagulation parameters, microvascular thrombus formation, organ damage parameters, and inflammatory cytokines in rats injected with LPS.

## Materials and methods

### Materials

Edoxaban tosylate was synthesized by Daiichi Sankyo Co., Ltd. (Tokyo, Japan) and dissolved in 9.2% cyclodextrin solution. LPS (*E. coli* 055:B5) was purchased from Sigma-Aldrich Co. LLC. (St. Louis, MO, USA). Enzygnost TAT micro and LPIA-ACE D-D dimer II were purchased from Siemens Healthcare Diagnostics (Tokyo, Japan) and LSI Medience Corporation (Tokyo, Japan), respectively. FUJI DRI-CHEM Slides (GOT/AST-PIII, GPT/ALT-PIII, BUN-PII, CRE-PIII) were obtained from FUJIFILM Medical Co., Ltd. (Tokyo, Japan). Procarta Cytokine Assay Kit was purchased from Panomics, Inc. (Fremont, CA, USA). ^125^I-fibrinogen (human) was obtained from PerkinElmer Japan Co., Ltd. (Yokohama, Japan).

### Animals

Male Slc:Wistar rats (10 weeks old when used) were purchased from Japan SLC (Hamamatsu, Japan) and acclimated for 1 week or more. Animal facilities, animal care, and study programs were in accordance with the in-house guideline of the Institutional Animal Care and Use Committee of Daiichi Sankyo Co., Ltd. Rats were housed 4 to 6 per cage at a temperature of 23 °C and humidity of 55% in a 12-h light–dark cycle (lighting from 7:00 to 19:00). Food and water were available ad libitum.

### LPS-induced microvascular thrombosis model

Under halothane anesthesia (halothane 1.5–2%, N_2_O 700 ml/min, O_2_ 300 ml/min), LPS (7.5 mg/kg, suspended in saline) was injected into the tail vein of rats (n = 6 or 7/group). For the sham group, saline was injected into the tail vein. Immediately after LPS injection, edoxaban was subcutaneously administered to the rats at doses of 0.3, 1, and 3 mg/kg. As the control, 9.2% cyclodextrin solution was subcutaneously administered. Two and six h after LPS injection, blood was collected from the jugular vein into a syringe containing 1/10 volume of 3.13% sodium citrate tribasic dehydrate. Platelet numbers in blood were counted, and then plasma was prepared by centrifugation of residual blood sample at 1500×*g* for 10 min at 4 °C. At the same time, blood for serum sample was also collected from the jugular vein and left at room temperature for 0.5–1 h to prepare serum. Then, the rats were killed by exsanguination under halothane anesthesia and liver and kidney were excised. Liver and kidney were fixed in 10% neutral buffered formalin for preparation of histopathological tissue specimens.

Platelet numbers were counted using an automatic counter MEK-6358 Celltac α (Nihon Kohden, Tokyo, Japan). To determine the coagulation status, plasma TAT concentration was measured using an enzyme-linked immunosorbent assay kit, Enzygnost TAT micro, according to the manufacturer’s protocol. Plasma D-dimer concentration was determined using a latex agglutination test LPIA-ACE D-D dimer II with ACL TOP500 (LSI Medience Corporation, Tokyo, Japan).

To determine the extent of organ damage, serum levels of aspartate aminotransferase (AST), alanine aminotransferase (ALT), blood urea nitrogen (BUN), and creatinine (CRE) were measured using FUJI DRI-CHEM Slides (GOT/AST-PIII, GPT/ALT-PIII, BUN-PII, CRE-PIII) with FUJI DRI-CHEM 7000 V (FUJIFILM Medical Co., Ltd., Tokyo, Japan).

Microvascular thrombus formation in the liver and kidneys was evaluated by the pathological analysis. Paraffin sections of the liver samples were stained with phosphotungstic acid-hematoxylin (PTAH). Ten fields were randomly selected per individual animal, and the numbers of fibrin thrombi formed in the hepatic sinusoids were counted using a 10 × eyepiece and a 40 × objective lens under blinded conditions. The total numbers of fibrin thrombi in the 10 fields were taken as the individual data of each rat.

In another set of experiment, mortality was assessed 24 h after LPS (7.5 mg/kg) treatment (n = 9–11/group). The doses of edoxaban were 0.1, 0.3, 1, and 3 mg/kg.

### Microvascular thrombus formation

Microvascular thrombus formation in organs was also evaluated using ^125^I-fibrinogen [14] as follows. Under halothane anesthesia, ^125^I-fibrinogen (66 kBq/kg) was injected into the jugular vein and 4 min later blood was collected as a pre-LPS blood sample (blood at 0 h). Five minutes after ^125^I-fibrinogen injection, LPS (7.5 mg/kg) was injected into the jugular vein and edoxaban (0.3, 1, and 3 mg/kg) was subcutaneously administered to the rats (n = 5/group). Blood samples were collected 6 h after LPS injection, and the animals were killed by exsanguination. The livers and kidneys were harvested. Radioactivities in blood and organ samples were measured with a gamma counter (Packard Cobra II model 5010, PerkinElmer Inc., Waltham, MA, USA). The residual radioactivity in the organs (% of radioactivity in blood at 0 h) was calculated by the following formula:

$$^{{{125}}} {\text{I-concentration in the organ (\%) = }}^{{{125}}} {\text{I in the organ/organ weight/}}^{{{125}}} {\text{I in blood at 0 h }} \times {100}$$

### Inflammatory cytokine production

LPS (5 mg/kg) was injected into the tail vein of rats as mentioned above (n = 5 or 7/group). The effects of edoxaban was evaluated at a dose of 10 mg/kg. Two and six h after LPS injection, blood was collected and plasma was prepared as mentioned above.

As inflammation cytokines, plasma levels of interleukin-6 (IL-6), tumor necrosis factor-α (TNF-α), and monocyte chemoattractant protein-1 (MCP-1) were determined using the Procarta Cytokine Assay Kit with Luminex 200 System (Luminex Corporation, Austin, TX, USA).

### Statistical analysis

Data are expressed as mean ± standard error of mean (SEM). The statistical analyses were performed using SAS System Release 8.2 (SAS Institute Inc., Cary, NC, USA). To compare difference between the sham and LPS groups, Student t-test or Welch t-test was performed after the equal or unequal variance was confirmed by F test. To compare difference between the LPS and edoxaban groups, Dunnett test was carried out. Mortality in the LPS and edoxaban groups were compared by Fisher's exact test. A *P* value of less than 0.05 (two-tailed) was considered to be a significant difference.

## Results

### Effects of edoxaban on coagulation markers and microvascular thrombus formation

**Fig. 1:**
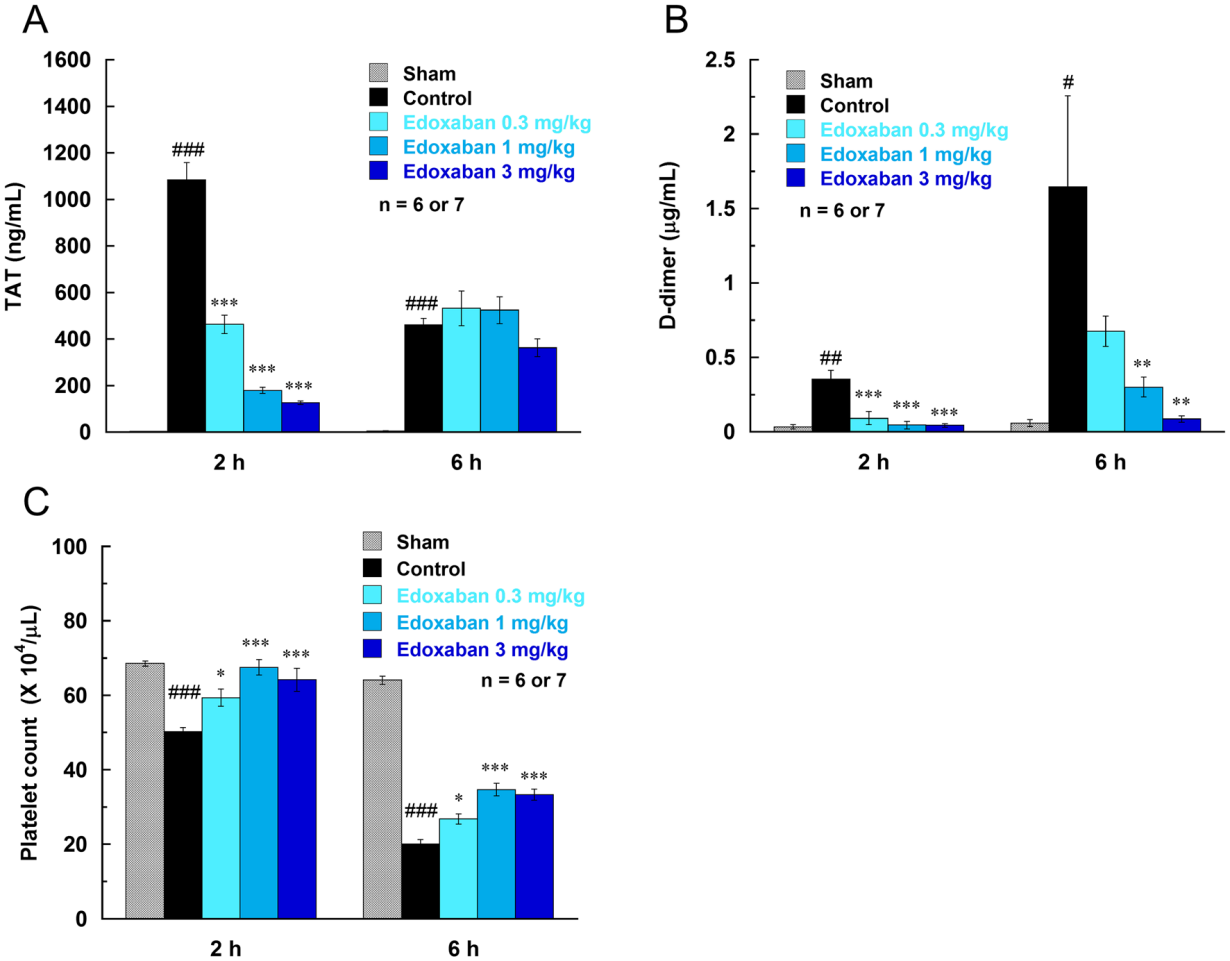
Effects of edoxaban on plasma concentrations of thrombin-antithrombin complex (TAT; **a**) and D-dimer (**b**) and platelet numbers (**c**) in rats injected with LPS (7.5 mg/kg, i.v.). Edoxaban was administered subcutaneously at doses of 0.3, 1, and 3 mg/kg immediately after the LPS injection. Blood was collected 2 and 6 h after the LPS injection. Sham was not treated with LPS. Control and Edoxaban were treated with LPS. Columns and bars represent mean ± SEM (n = 6 or 7). ^#^*Ρ* < 0.05, ^##^*Ρ* < 0.01, ^###^*Ρ* < 0.001 compared with the sham group. *Ρ < 0.05, **Ρ < 0.01, ***Ρ < 0.001 compared with the LPS control group

By the intravenous injection of LPS (7.5 mg/kg), plasma TAT and D-dimer concentrations significantly increased, and platelet numbers decreased (Fig. 1). The increase in TAT level was rapid, maximum at 2 h after LPS injection, and then decreased at 6 h. The increase in D-dimer and decrease in platelet numbers were more pronounced at 6 h than at 2 h after LPS injection. These data indicates that LPS induced the activation of the coagulation pathway, and subsequently resulted in the consumption of platelets and production of the fibrin degradation product D-dimer. Edoxaban attenuated the changes in these coagulation markers, suppressed the increase in TAT and D-dimer and decrease in platelet numbers in a dose-dependent manner.

**Fig. 2:**
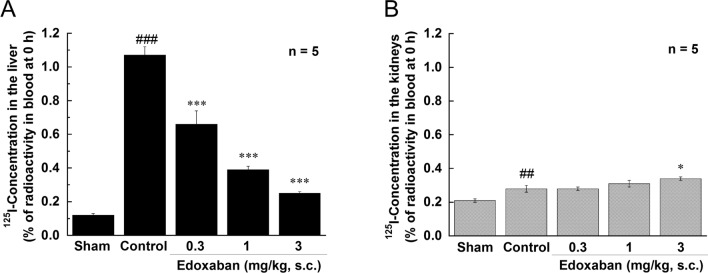
Effect of edoxaban on fibrin deposition in organs in rats injected with LPS (7.5 mg/kg, i.v.). Fibrin depositions were induced by the intravenous injection of LPS (7.5 mg/kg) and determined using ^125^I-labeled fibrinogen. The residual radioactivity in the organs 6 h after the LPS injection (% of radioactivity in blood at 0 h) was calculated by the following formula: ^125^I-concentration in the organ (%) = ^125^I in the organ/organ weight/^125^I in blood at 0 h × 100. Edoxaban was administered subcutaneously at doses of 0.3, 1, and 3 mg/kg immediately after the LPS injection. Sham was not treated with LPS. Control and Edoxaban were treated with LPS. Columns and bars represent mean ± SEM (n = 5). ^##^*Ρ* < 0.01, ^###^*Ρ* < 0.001 compared with the sham group. *Ρ < 0.05, ***Ρ < 0.001 compared with the LPS control group

As an indicator of fibrin deposition, residual radioactivities in the liver and kidney were measured in rats injected with ^125^I-fibrinogen. LPS significantly increased the residual radioactivity in the liver 6 h after LPS injection (Fig. 2). Edoxaban (0.3, 1, and 3 mg/kg, s.c.) dose-dependently and significantly suppressed the increase in the liver radioactivity induced by LPS. There was a mild increase in residual radiological activity in the kidney, however edoxaban had no inhibitory effect on it.

**Fig.3:**
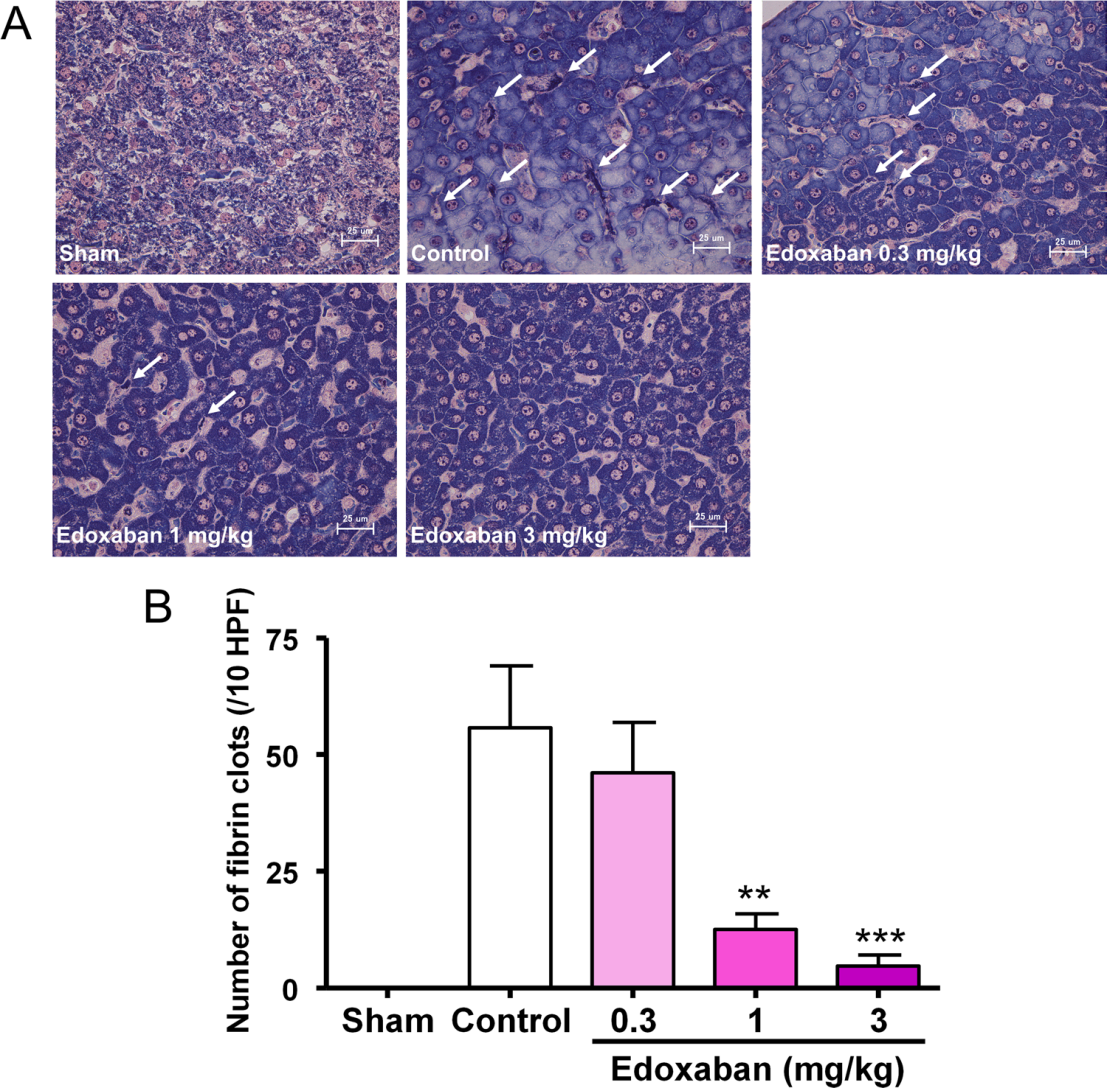
Effect of edoxaban on fibrin clot formation in sinusoids of the livers in rats injected with LPS (7.5 mg/kg, i.v.). Edoxaban was administered subcutaneously at doses of 0.3, 1, and 3 mg/kg immediately after the LPS injection. The livers were harvested 6 h after LPS injection. **a** Photomicrographs of the livers (PTAH staining, high power view). Arrows indicate fibrin clots in sinusoids. **b** Effect of edoxaban on the numbers of fibrin clots. Sham was not treated with LPS. Control and Edoxaban were treated with LPS. HPF, high power field. Columns and bars represent mean ± SEM (n = 6 or 7). **Ρ < 0.01, ***Ρ < 0.001 vs control

The histopathological analysis of the livers of LPS-treated rats also showed the formation of microvascular thrombus and leukocyte infiltration in sinusoids (Fig. 3). There were no fibrin thrombus in the kidneys. Treatment with edoxaban attenuated the microvascular thrombus formation in the liver.

### Effects on organ damage markers, mortality, and inflammatory cytokines

**Fig. 4:**
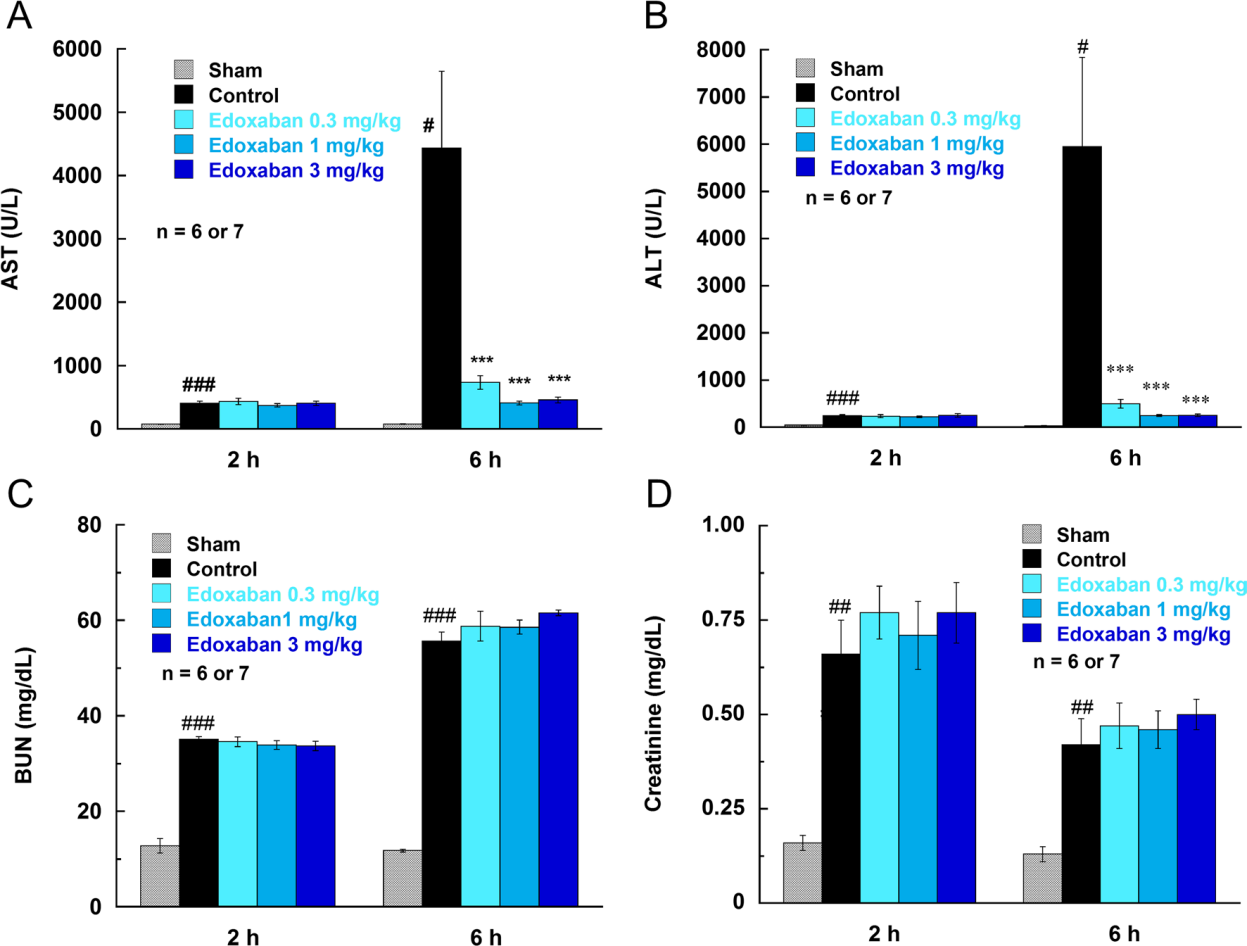
Effects of edoxaban on serum levels of aspartate aminotransferase (AST; **a**), alanine aminotransferase (ALT; **b**), blood urea nitrogen (BUN; **c**), and creatinine (**d**) in rats injected with LPS (7.5 mg/kg, i.v.). Edoxaban was administered subcutaneously at doses of 0.3, 1, and 3 mg/kg immediately after the LPS injection. Blood was collected 2 and 6 h after the LPS injection. Sham was not treated with LPS. Control and Edoxaban were treated with LPS. Columns and bars represent mean ± SEM (n = 6 or 7). ^#^*Ρ* < 0.05, ^##^*Ρ* < 0.01, ^###^*Ρ* < 0.001 compared with the sham group. ***Ρ < 0.001 compared with the LPS control group

LPS (7.5 mg/kg, i.v.) elevated the liver damage markers, AST and ALT, and the kidney damage markers, BUN and CRE (Fig. 4). The elevations of AST, ALT, and BUN were pronounced 6 h after LPS injection, which was similar timing of microvascular thrombus formation. Edoxaban suppressed the elevations of AST and ALT more than 90% consisting with its inhibitory effects on the microvascular thrombus formation in the liver, whereas edoxaban had no effect on BUN and CRE. The histopathological analysis showed that edoxaban had a tendency to reduce necrotic changes in hepatocytes (data not shown). Edoxaban significantly reduced mortality 24 h after LPS injection (Table 1).

**Table 1:** Effect of edoxaban on the mortality assessed 24 h after LPS treatment (7.5 mg/kg, i.v.) in rats

Edoxaban (mg/kg, s.c.)	Mortality (dead/total)	*P* (vs. Control)
Control	10/10	
0.1	9/10	1.0000
0.3	2/9	0.0007
1	6/11	0.0351
3	1/10	0.0001

Mortality in the control and edoxaban groups were compared by Fisher's exact test

**Fig. 5:**
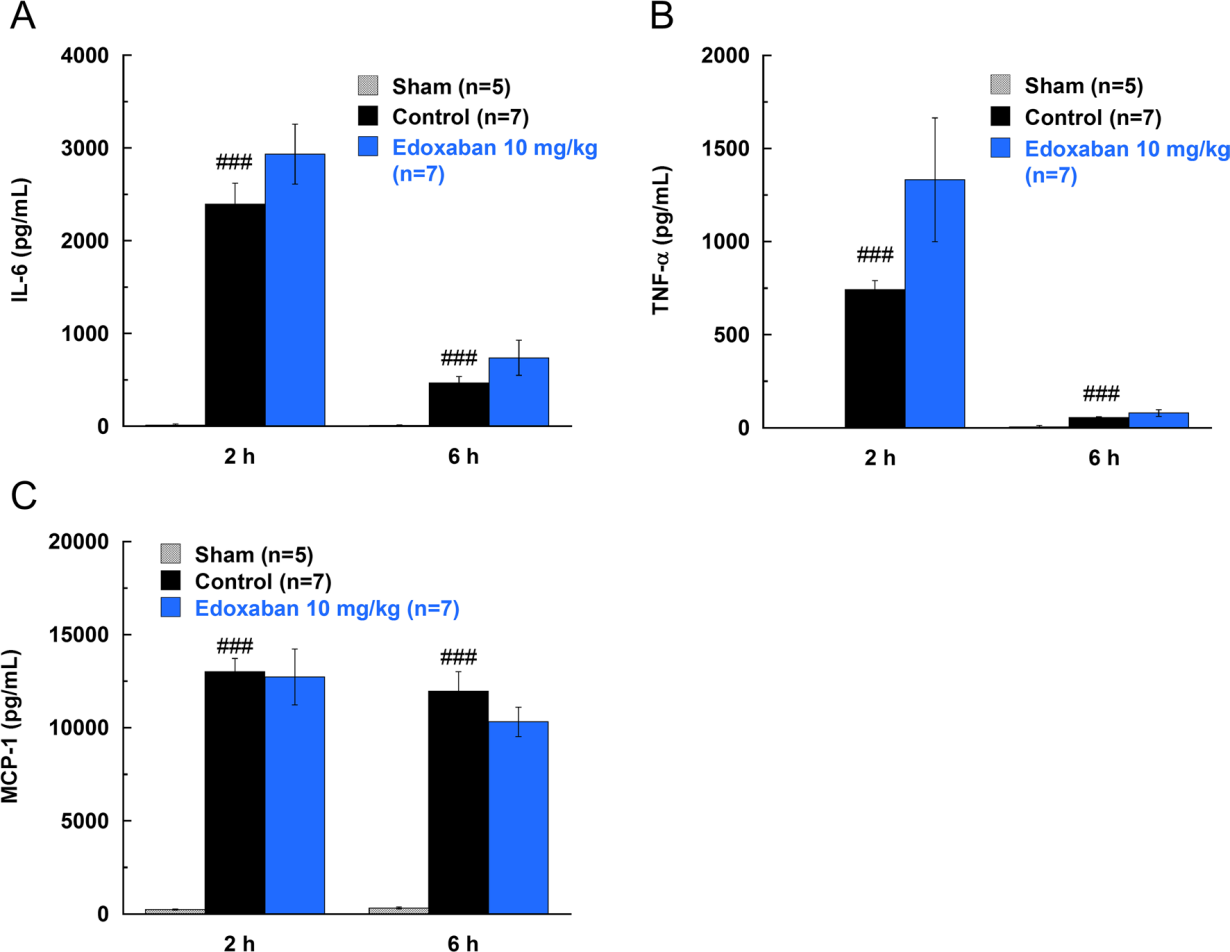
Effects of edoxaban on the plasma levels of inflammatory cytokines in rats injected with LPS (5 mg/kg, i.v). **a** Interleukin-6 (IL-6). **b** Tumor necrosis factor-α (TNF-α), **c** Monocyte chemoattractant protein-1 (MCP-1). Edoxaban was administered subcutaneously at a dose of 10 mg/kg immediately after the LPS injection. Blood was collected 2 and 6 h after the LPS injection. Sham was not treated with LPS. Control and Edoxaban were treated with LPS. Columns and bars represent mean ± SEM (n = 5 or 7). ^###^*Ρ* < 0.001 compared with the sham group

In rats treated with 5 mg/kg LPS, plasma levels of inflammatory cytokines, IL-6, TNF-α, and MCP-1, were elevated (Fig. 5). Edoxaban (10 mg/kg, s.c.) did not inhibit the production of these inflammatory cytokines.

## Discussion

In this study, we have chosen LPS as an inducer of infection-associated thrombosis in rats. LPS is the most frequently used inducer to mimic coagulopathy associated with infection in animal models [3]. In accordance with previous studies [6–9], intravenous injection of LPS induced the production of inflammatory cytokines, the activation of coagulation pathway (increased TAT and D-dimer, decreased platelet numbers), the formation of microvascular thrombus, and the liver and kidney damages in our model. In this LPS-induced thrombosis model, the direct FXa inhibitor, edoxaban, significantly inhibited the hypercoagulation, the fibrin deposition in the liver, and the elevation of the liver damage parameters in a dose-dependent manner. Furthermore, edoxaban significantly reduced mortality caused by LPS. However, edoxaban did not suppressed the elevation of inflammatory cytokines and the kidney damage. As far as we know, this is the first study which shows the inhibitory effect of DOAC on infection-associated thrombosis in animal models.

The elevation of inflammatory cytokines and TAT peaked at an early time point 2 h after LPS injection, then, elevation of D-dimer, fibrin deposits formation, and organ damages were observed 6 h after LPS injection. The study by Pawlinski et al. [6] suggests that binding of LPS to monocytes/macrophages, platelets or endothelial cells results in a procoagulant state through directly activating inflammatory responses concurrently with coagulation factors like monocyte-derived tissue factor. Thus, in our LPS model, coagulation activation could occur parallel to or as a result of the inflammatory response, leading to thrombus formation and subsequent liver damage caused by circulatory disturbance. Edoxaban inhibited FXa in the coagulation pathway, reduced fibrin deposition, and necrotic changes in the liver. On the other hand, in the kidneys, the increase in the residual radioactivity was less than that in the liver and no fibrin deposit formation was observed. Moreover, edoxaban had no effects on the levels of renal damage markers, BUN and CRE. Therefore, the damages in the kidneys under the present conditions were tubular damages directly induced by LPS, but not caused by thrombus formation. Schneider previously showed that deposition of ^125^I-fibrin is more prominent in the liver than in the kidneys [14], suggesting that the liver is more prone to cause microvascular thrombus formation and more susceptible to the secondary damage due to impaired circulation at least under the experimental conditions.

Some studies determined the effects of anticoagulants, like low molecular weight heparin, tissue factor pathway inhibitor, and hirudin, on fibrin deposition induced by intravenous administration of LPS [6, 8, 9]. These studies showed that the anticoagulants significantly attenuates hypercoagulation and fibrin deposition induced by LPS. Especially, in consistent with our study, hirudin, a thrombin inhibitor, reduces LPS-induced fibrin deposition in the liver and prolongs the survival time, but has no effect on the induction of inflammatory cytokines [6]. Protease activated receptors (PARs) are known to play an important role in the inflammatory response mediate by the coagulation activation [15]. FXa and thrombin mainly activate PAR-1 and PAR-2, respectively, and cause cytokine production. Since edoxaban or hirudin solely did not reduce inflammation, the inhibition of a single coagulation factor, like FXa or thrombin, could not attenuate inflammation mediated by the coagulation activation. These data suggest that there may be a redundant pathways between coagulation and inflammation and both PAR-1 and PAR-2 may contribute to the crosstalk [6].

Recently, the high risks of both venous and arterial thrombosis in patients with COVID-19 are emerging [16, 17]. Thrombosis might be contributed to the mortality in these patients, because patients with high levels of D-dimer at admission results in the higher rate of death [18] and the anticoagulant therapy is suggested to improve survival in in-hospital COVID-19 patients [19]. Marongiu et al. reported a possible mechanism inducing thrombosis in patients with COVID-19 [20]. After novel coronavirus infection, monocytes and inflammatory cytokines can provoke endothelial damage and blood coagulation activation. Although *E. coli* (LPS) and novel coronavirus are different as pathogens, the mechanisms to induced thrombosis may be similar. Thus, we speculate that there would be benefit with edoxaban in COVID-19 patients, probably due to inhibition of thrombosis and subsequent organ damage and death.

In conclusions, edoxaban significantly inhibited the activation of coagulation, the formation of microvascular thrombus in the liver and the liver damage, and reduced mortality in rats injected with LPS. Whereas, edoxaban has no beneficial effects on the kidney damage and the production of inflammatory cytokines. These results suggest that the FXa inhibition by edoxaban might be a beneficial therapy for the management of infection-associated thrombosis.
